# Editorial: The tumor microenvironment and immunotherapy for head and neck tumors

**DOI:** 10.3389/fonc.2025.1606787

**Published:** 2025-06-04

**Authors:** Yifan Jiang, Wenyi Yang, Jingzhou Hu, Lei Tao

**Affiliations:** ^1^ Department of Otolaryngology, Eye & ENT Hospital, Fudan University, Shanghai, China; ^2^ Department of Oral Maxillofacial & Head and Neck Oncology, Shanghai Ninth People’s Hospital, Shanghai Jiao Tong University School of Medicine, Shanghai, China

**Keywords:** head and neck tumor, tumor microenvironment, immunotherapy, immune checkpoint inhibitors, immune evasion

Head and neck tumors represent a highly heterogeneous group of malignancies, both biologically and anatomically, posing significant challenges for clinical research and therapeutic optimization. This heterogeneity is particularly relevant in the context of immunotherapy, where the diverse immune landscapes profoundly influence treatment outcomes. Central to this dynamic is the tumor microenvironment (TME), which plays a pivotal role in tumor progression, immune evasion, and response to immunotherapy (1). Despite the efficacy of immune checkpoint inhibitors (ICIs), such as PD-1/PD-L1 blockade, in a subset of head and neck squamous cell carcinoma (HNSCC) patients, therapeutic resistance and immune evasion remain major clinical challenges. This Research Topic brings together several studies investigating key TME mechanisms, immune evasion, novel immunotherapy approaches, and clinical translation.

The immune composition of HNSCC varies significantly depending on factors such as HPV status (2, 3) and tumor location. Shivarudrappa et al. investigated immune dynamics in murine models expressing HPV16 E6/E7 and identified two distinct phenotypes: one exhibiting complete tumor eradication (C-225) and the other showing progressive growth (C-100). Intriguingly, the latter group displayed a higher CD8+ T cell infiltration but a pronounced functional exhaustion, marked by an elevated expression of PD-1 and LAG-3. This paradox highlights the critical role of immune checkpoint activation and myeloid-derived suppressor cell (MDSC) enrichment in driving immune evasion, even in the context of robust T cell infiltration. Moreover, tertiary lymphoid structures (TLS) are emerging as critical components of anti-tumor immunity (4, 5). Wu et al. developed a TLS scoring system and revealed that TLS presence correlated with PD-1+CXCL13+CD8+ T cell activity and improved immune responses in HNSCC patients. A case report by Jiang et al. examined the phenomenon of a “dissociated response” to immunotherapy, in which certain tumor sites regress while others progress. This observation underscores the spatial heterogeneity of the TME and may influence treatment strategy in advanced head and neck cancer.

Head and neck tumors frequently exhibit immune escape mechanisms that limit treatment efficacy. The identification of reliable biomarkers is crucial for predicting tumor progression and response to immunotherapy. In this Research Topic, He et al. investigated BANF1, which modulates immune infiltration and is associated with poor prognosis, likely due to its role in impairing immune cell recruitment within the TME of HNSCC. Another study by Qin et al. developed a disulfidptosis-related gene risk model (SLC3A2, NUBPL, ACTB, DSTN) for HNSCC prognostic stratification. Notably, DSTN was significantly upregulated in HNSCC and was shown to promote tumor progression, whereas its knockdown inhibited tumor growth, migration, and invasion—highlighting its potential as both a prognostic and therapeutic biomarker. Moreover, Yang et al. identified DLX6 as a novel prognostic biomarker for nasopharyngeal carcinoma (NPC). DLX6 expression was correlated with poor prognosis and was found to promote cell proliferation, invasion, and migration. It also impacted the immune landscape of NPC, suggesting its role in metastasis and immune modulation. Qin et al. explored the function of IRX5 in papillary thyroid carcinoma and found that its expression promoted macrophage polarization toward an M2 phenotype, thereby exacerbating immune suppression within the TME.

In addition to immune cell infiltration, other factors, such as stromal interactions and metabolic regulators, play a key role in tumor behavior. In this context, Pan et al. reviewed the role of neck adipose tissue (NAT) in head and neck cancer, highlighting its influence on cervical lymph node metastasis. Through the secretion of adipokines such as leptin, adiponectin, and interleukin-6, NAT may promote metastatic progression. A more profound understanding of the role of NAT and the interactions between cancer cells and adipocytes may inform new therapeutic strategies, particularly through targeted therapy or metabolic intervention.

Recent developments in head and neck cancer treatment highlight the growing importance of immunotherapy combinations, particularly in the development of personalized, organ-preserving approaches. Wu et al. conducted a retrospective cohort study involving 20 patients with locally advanced hypopharyngeal cancer (LAHPC) to evaluate the efficacy of neoadjuvant chemotherapy combined with ICIs. Their findings revealed a 50% pathological complete response rate at the primary tumor site and an impressive 95% laryngeal preservation rate. While these outcomes are encouraging, longer-term follow-up remains necessary to evaluate the sustained survival benefit and functional preservation. Gao et al. reported two cases of *de novo* metastatic NPC (dmNPC), in which chemotherapy combined with a PD-1 inhibitor—without locoregional radiotherapy—achieved durable remission. These findings suggest that carefully selected patients may benefit from radiation-sparing approaches, potentially avoiding treatment-related toxicities while maintaining therapeutic efficacy. In another case report, Wang et al. described a complete remission in a patient with NPC and extensive bone marrow metastases following a stepwise, individualized immunotherapy-based combination regimen. This underscores the value of flexible, multidisciplinary strategies tailored to disease burden and treatment tolerance. Furthermore, Shan et al. found that early-onset head and neck squamous cell carcinoma (HNSCC) may harbor a distinct tumor microenvironment characterized by enhanced immune suppression, potentially indicating greater sensitivity to immunotherapeutic interventions in this subgroup.

Innovative therapeutic approaches are being explored in head and neck tumors. Sun et al. reviewed advances in CAR-T cell therapy for thyroid cancer. CAR-T cells constructed with antigens such as TSHR, ICAM-1, GFRα4, B7-H3, and CEA have demonstrated preclinical anti-tumor activity. However, only a few have progressed to clinical trials, with results still pending. Further optimization of CAR constructs, enhancement of T cell activation, and strategies to overcome the immunosuppressive TME remain critical challenges. Meanwhile, nanomedicine is an emerging field that offers new avenues for HNSCC treatment. In a review by Li et al., nanodrug delivery systems are highlighted for their ability to enhance radiotherapy sensitivity and optimize immune targeting, thus expanding the therapeutic options available for HNSCC. Turner et al. summarized recent advances in therapeutic options for follicular-derived thyroid cancer. Despite progress with kinase inhibitors and ICIs, radioiodine-refractory differentiated thyroid cancers and anaplastic thyroid cancers remain therapeutic challenges. Strategies targeting M2 macrophages, dendritic cells, and NK cells may hold promise for overcoming resistance. While many of these strategies are still in the experimental or early clinical stages, their development underscores the rapid evolution of immunotherapy in head and neck tumors.

In clinical practice, the introduction of ICIs has revolutionized the treatment paradigm for HNSCC. Landmark trials such as CheckMate 141 (6) and KEYNOTE-048 (7) have led to the incorporation of PD-1 inhibitors in treatment guidelines for recurrent or metastatic HNSCC, establishing ICIs as a cornerstone of therapy in this setting. The demonstrated efficacy has catalyzed the systematic evaluation of ICIs in earlier stages of disease, with locally advanced HNSCC (LA-HNSCC) representing a major therapeutic challenge under investigation. The KEYNOTE-689 trial (8)—which evaluated pembrolizumab as both neoadjuvant and adjuvant therapy in combination with standard of care (radiotherapy ± cisplatin) for previously untreated, resectable LA-HNSCC—reportedly met its primary endpoint and has the potential to be practice-changing.

In the future, realizing the full potential of immunotherapy in this heterogeneous disease spectrum will require sustained multidisciplinary collaboration, well-designed clinical trials, and the integration of advanced technologies such as single-cell (9) and spatial omics (10). Ultimately, bridging the gap between mechanistic insights and clinical application will be crucial for improving outcomes for patients with head and neck tumors.
